# Primary undifferentiated embryonal sarcoma of the liver mistaken for hydatid disease

**DOI:** 10.1186/1477-7819-8-58

**Published:** 2010-07-09

**Authors:** Walid Faraj, Deborah Mukherji, Nadim El Majzoub, Ali Shamseddine, Achraf Shamseddine, Mohamed Khalife

**Affiliations:** 1 Department of Surgery, HPB and liver transplantation unit, American University of Beirut Medical Center, Beirut, Lebanon; 2 Department of Internal medicine, Oncology unit, American University of Beirut Medical Center, Beirut, Lebanon

## Abstract

Primary undifferentiated embryonal sarcoma of the liver is a rare tumor with a peak incidence between the ages of 6 and 10 years. We report a case of a primary hepatic undifferentiated embryonal sarcoma arising in a 21-year-old male mistaken for hydatid disease of the liver. The rapid recurrence of this tumor along the site of attempted percutaneous drainage illustrates some important management points regarding this malignancy.

## Introduction

Primary undifferentiated embryonal sarcoma (UES) of the liver is a rare and highly malignant neoplasm of mesenchymal origin. The majority of primary hepatic malignancies are carcinomas with primary hepatic sarcomas representing between 0.1% and 2% of primary hepatic cancers [1]. UES of the liver is most commonly seen arising in children with a peak incidence between 6 and 10 years but can arise in adults [2].

Our institution is a leading tertiary referral centre for the treatment of hepatic malignancies in the region. From 1998 to 2009, 215 adult patients were diagnosed with primary hepatic malignancies at our institution, 4 of which were diagnosed with primary hepatic sarcoma (1.8%). Three cases were primary hepatic leiomyosarcomas (LMS) and one case was primary UES of the liver mistaken for hydatid disease in a 21-year-old male patient. Hydatid disease is endemic in the Middle East and is top of the differential diagnosis for a cystic liver lesion presenting in this age group.

## Case Report

A 21 year old male patient was referred to our institution following unsuccessful surgery for presumed hydatid disease of the right lobe of the liver. The initial operation was performed in a district hospital; an open approach was performed and an attempt to drain/resect the lesion failed. The operation was aborted and a percutaneous drain was inserted at the site of surgery. The patient was in hospital for 2 weeks with no improvement. The family decided to transfer him to our institution which is a tertiary care center specialized in hepatobiliary surgery.

On admission, the patient was in respiratory distress with severe abdominal pain, distension and lower limb edema. The alpha-fetoprotein (AFP) at the time of diagnosis was 3.6 U/mL (range 1-9), carcinoembryonic Ag (CEA) 2.2 ng/mL (range 0-4) and human chorionic gonadotropin (Beta- HCG) 3.6 mlU/mL (< = 4). The laboratory tests included INR median 1.4 (range 0.9-1.1), serum bilirubin (Total) 1.4 mg/dL (range 0-1.2), serum aspartate aminotransferase (AST) 113 IU/L (range 0-50), gamma-glutamyl transferase 95 IU/L (range 0-50), creatinine 0.6 mg/dL (range 0.5-1.2), platelet count 680 × 10^9^/L (range 150-400) and haemoglobin 11 g/dl (range 13-18). ELISA test was negative for hydatid disease Serology was negative for hepatitis B, C and HIV.

**Figure 1:**
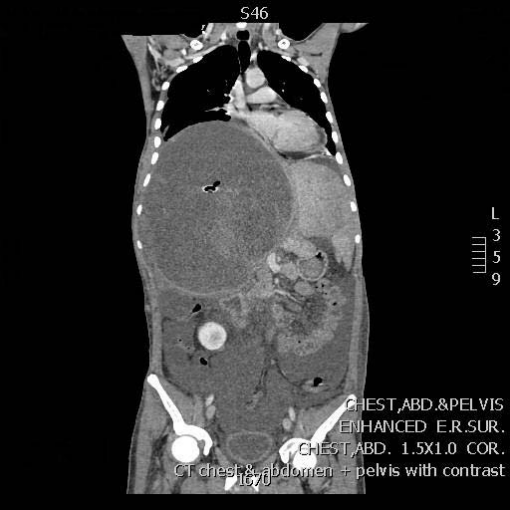
**A computed tomography (CT) scan of the abdomen with a large lesion occupying the right hepatic lobe**.

A computed tomography (CT) scan of the abdomen revealed a large, well defined lesion with intracystic septations occupying the right hepatic lobe measuring 22 × 19 × 23.6 cm containing the percutaneous drain (Figure 1). Significant abdominal ascites was noted.

**Figure 2:**
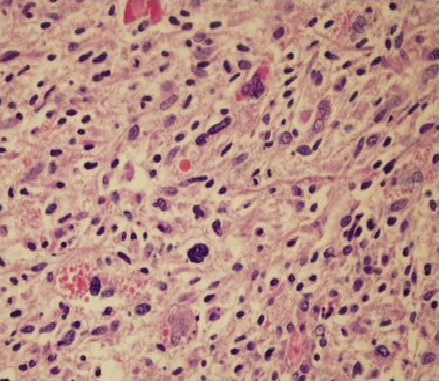
**Histopathology of the UES of the liver with irregular spindle cells showing moderate pleomorphism and brisk mitotic activity**.

**Figure 3:**
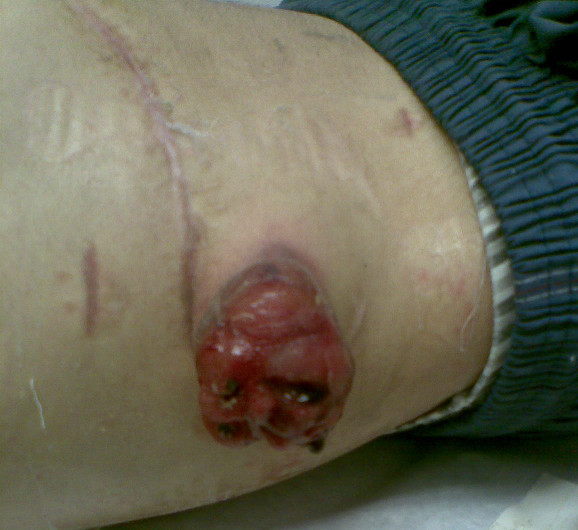
**Intra-abdominal lesion extending through the drain site 5 cm outside the skin**.

A technically challenging extended right hepatectomy was performed. Histopathology revealed an unusual neoplasm composed of irregular spindle cells showing moderate pleomorphism and brisk mitotic activity (Figure 2). Immunohistochemical studies showed positive staining for vimentin and a positive cytokeratin AE1/3. Diagnosis was made of a stage III undifferentiated embryonic sarcoma (UES). The patient was discharged home day 16 post-resection. Adjuvant chemotherapy was recommended and the patient elected to be treated in his native country. Three weeks post-discharge he re-presented to our institution with a fungating abdominal wall mass at the site of previous percutaneous drain insertion. CT scan revealed a new lesion in segment II and an intra-abdominal lesion extending through the drain site 5 cm outside the skin (Figure 3). The skin lesion was resected and histopathological examination revealed metastatic UES. He elected to go back to his country of origin where he received adjuvant chemotherapy which was ifosfamide plus etoposide alternating with actinomycin-D, vincristine every 3 weeks for 6 months. He is currently 5 months post-resection and a recent CT scan showed no evidence of disease recurrence.

## Discussion

Diagnosis of primary hepatic sarcoma is challenging due to the lack of specific presenting symptoms, lack of serological markers, non-specific findings on radiological imaging and the rarity of the disease. Pachera et al recently reviewed the literature on primary hepatic UES and found that in the past 50 years only 51 cases have been reported in patients older than 15 years [3]. UES often show a misleading cystic appearance on CT and magnetic resonance imaging (MRI) in contrast to a predominantly solid appearance on ultrasound [4]. This finding may help to avoid attempts at drainage as in the case we have presented. In their literature review, Pachera et al found that the diagnosis of UES was delayed in 12 cases (23.5%) where the presentation with large cystic hepatic mass was suggestive of a benign lesion. There have been previous case reports of hepatic UES being mistaken for hydatid disease [5-8] however this is the first report of a case in which the diagnosis was made after attempted percutaneous drainage with evidence of rapid track-seeding and progression post-resection.

Hydatid disease is a common pathology in our region and the surgical options are numerous. Surgeons may opt to remove the cysts through an open approach or laparoscopic approach. In either approach, it should be decided whether a radical path or a more conservative one should be taken. With regards to open surgery, several recent studies have shown that the radical approach is associated with lower rates of recurrence, fewer complications such as bile leak and decreased mortality. Percutaneous drainage of hydatid disease is another option, but we try to avoid this due to the risk of intra-abdominal spillage.

In our institution, both open and laparascopic procedures are performed and the choice of approach depends on the size and location of the hydatid lesion [9-11].

Complete resection followed by adjuvant chemotherapy is the current standard of care for hepatic UES, however due to the rarity of the disease, limited data exists and treatment remains largely empirical. Positive resection margins and spontaneous or iatrogenic rupture of the tumour are associated with early recurrence and death [3]. Lenze et al reviewed treatment outcomes for 68 patients over the age of 15 years and found a median survival of 29 months. Patients who underwent complete resection followed by adjuvant chemotherapy had significantly better survival compared with patients who underwent surgical resection alone. As expected, incomplete resection was associated with poorer outcome [12].

There have been three reports of liver transplantation for UES in children [13-15] however the use of liver transplantation for primary hepatic sarcoma in adults is controversial. There are no reports of liver transplantation for UES in adults and outcomes of liver transplantation for other histological subtypes such as hepatic angiosarcoma and LMS have been disappointing [16,17].

The case we have presented demonstrates the propensity of this tumour to rapidly recur along a percutaneous track however tumour control was achieved with a second radical resection followed by adjuvant chemotherapy.

## Conclusion

In summary, this case illustrates some important aspects of this rare disease:

1. The discrepancy between CT and ultrasound appearances of this lesion is a key factor that should raise the index of suspicion when noted.

2. Due to the propensity of this lesion to seed along a percutaneous track, percutaneous biopsy or percutaneous drainage should not be attempted.

3. Complete resection must be attempted including resection of any potential percutaneous track followed by adjuvant chemotherapy.

4. If a percutaneous track cannot be resected, radiotherapy should be considered to reduce the risk of recurrence.

## Competing interests

The authors declare that they have no competing interests.

## Authors' contributions

WF drafted the manuscript, NEM and DM participated in the design of the study, AcS assisted with the collection of data and conceived of the study, MK and AlS participated in the design and coordination of the study. All authors read and approved the final manuscript.

## Consent

Written informed consent was obtained from the patient for publication of this case report and accompanying images. A copy of the written consent is available for review by the Editor-in-Chief of this journal.
